# Enhanced Landslide Monitoring in Complex Mountain Terrain Using Distributed Scatterer InSAR and Phase Optimization: A Case Study in Zhenxiong, China

**DOI:** 10.3390/s26020430

**Published:** 2026-01-09

**Authors:** Jingyuan Liang, Bohui Tang, Menghua Li, Fangliang Cai, Lei Wei, Cheng Huang

**Affiliations:** 1 Faculty of Land Resources Engineering, Kunming University of Science and Technology, Kunming 650093, China; 20232101041@stu.kust.edu.cn (J.L.); menghuali@kust.edu.cn (M.L.); caifangliang@stu.kust.edu.cn (F.C.); yndzhjjcy@163.com (L.W.); hch2377@163.com (C.H.); 2 Yunnan Key Laboratory of Quantitative Remote Sensing, Kunming 650093, China; 3 Yunnan International Joint Laboratory for Integrated Sky-Ground Intelligent Monitoring of Mountain Hazards, Kunming 650093, China; 4 State Key Laboratory of Resources and Environmental Information System, Institute of Geographic Sciences and Natural Resources Research, Chinese Academy of Sciences, Beijing 100101, China; 5 Yunnan Institute of Geo-Environment Monitoring, Kunming 650216, China

**Keywords:** Distributed Scatterer InSAR (DS-InSAR), sequential estimation, Expectation-Maximization Inversion (EMI), polarimetric SAR, phase optimization

## Abstract

**Highlights:**

**What are the main findings?**

- This study applies the SETP-EMI method for the first time to plateau mountainous regions with dense vegetation, demonstrating its ability to overcome severe coherence loss.
- The integrated DS-InSAR framework significantly improves distributed scatterer density, phase stability, and deformation continuity compared with PS-InSAR and SBAS-InSAR.

**What are the implications of the main findings?**

- The demonstrated performance of SETP-EMI in challenging high-altitude, vegetation-covered terrain indicates its strong potential for large-scale geohazard monitoring in complex mountainous environments.
- The method provides an effective technical route for enhancing early-warning capability of landslides where conventional InSAR approaches typically fail due to low coherence.

**Abstract:**

Landslide deformation monitoring plays a critical role in geohazard prevention and risk mitigation in mountainous regions, where timely and reliable deformation information is essential for early warning and disaster management. Monitoring landslide deformation in mountainous areas remains a persistent challenge, largely due to rugged topography, dense vegetation cover, and low interferometric coherence—factors that substantially limit the effectiveness of conventional InSAR methods. To address these issues, this study aims to develop a robust time-series InSAR framework for enhancing deformation detection and measurement density under low-coherence conditions in complex mountainous terrain, and accordingly introduces the Sequential Estimation and Total Power-Enhanced Expectation–Maximization Inversion (SETP-EMI) approach, which integrates dual-polarization Sentinel-1 SAR time series within a recursive estimation framework, augmented by polarimetric coherence optimization. This methodology allows for dynamic assimilation of SAR data, improves phase quality under low-coherence conditions, and enhances the extraction of distributed scatterers (DS). When applied to Zhenxiong County, Yunnan Province—a region prone to geohazards with complex terrain—the SETP-EMI method achieved a landslide detection rate of 94.1%. It also generated approximately 2.49 million measurement points, surpassing PS-InSAR and SBAS-InSAR results by factors of 22.5 and 3.2, respectively. Validation against ground-based leveling data confirmed the method’s high accuracy and robustness, yielding a standard deviation of 5.21 mm/year. This study demonstrates that the SETP-EMI method, integrated within a DS-InSAR framework, effectively overcomes coherence loss in densely vegetated plateau regions, improving landslide monitoring and early-warning capabilities in complex mountainous terrain.

## 1. Introduction

Landslides represent one of the most widespread and destructive geohazards, posing severe threats to human safety, infrastructure stability, and socio-economic development, particularly in mountainous regions with diverse and fragile geological environments [1]. Beyond endangering lives and property, the unpredictability and complexity of landslides often limit the effectiveness of conventional ground-based monitoring systems in providing timely and comprehensive early warning information [2,3]. Consequently, for plateau and highland mountainous regions characterized by intricate and dynamic geological conditions, establishing a geohazard monitoring and early warning framework with extensive spatial coverage, high precision, and rapid responsiveness is an urgent requirement to reduce casualties, mitigate economic losses, and safeguard infrastructure. Within this context, rapid and effective identification of potential landslide hazards over large areas has become a central focus of geohazard research and disaster prevention.

Currently, commonly employed landslide identification approaches include remote sensing carried out by optical satellites [4] and by unmanned aerial vehicles (UAVs) [5], airborne LiDAR surveys [6], and Interferometric Synthetic Aperture Radar (InSAR) monitoring [7]. Optical remote sensing allows rapid, large-area observation but cannot capture ongoing ground deformation or measure deformation rates accurately. UAV remote sensing provides high-resolution data and can be deployed quickly for emergency monitoring, yet it is limited in achieving sustained, large-scale, or periodic observations. Airborne LiDAR offers high-precision measurements but is constrained by high operational costs and complex data processing. In contrast, InSAR enables all-weather, wide-area monitoring of surface deformation, making it particularly suitable for landslide detection in mountainous regions where traditional techniques face limitations due to complex terrain and dense vegetation.

Due to its ability to monitor surface deformation with high accuracy across large regions under all-weather and day–night conditions, Interferometric Synthetic Aperture Radar (InSAR) has been widely applied to the detection and monitoring of unstable slopes and actively deforming landslides [8,9]. Time-series Differential InSAR (D-InSAR) has been extensively utilized for early detection, continuous monitoring, pre-failure deformation tracking, and the examination of landslide dynamics [10,11,12,13], thereby enhancing geohazard surveillance and early-warning capabilities. However, conventional Persistent Scatterer Interferometry (PSI) techniques rely on single-master, single-polarization frameworks and primarily target high-amplitude, phase-stable scatterers, such as anthropogenic structures. In complex mountainous or densely vegetated regions, natural scatterers typically exhibit low backscatter and unstable phase, limiting the number of persistent scatterers and reducing the reliability of surface deformation measurements. To overcome these limitations, Distributed Scatterer InSAR (DS-InSAR) has been developed, which enhances phase estimation accuracy, mitigates decorrelation effects, and increases the density of coherent scatterers, thereby providing a more effective approach for time-series deformation analysis in heterogeneous and challenging environments. These inherent challenges highlight the need for advanced InSAR processing frameworks capable of handling low-coherence, complex terrain, and multi-polarization information—motivating the development of the SETP-EMI method presented in this study.

Owing to the distinct sensitivities of polarization channels to diverse scattering mechanisms and geometric features of ground objects, notable discrepancies in interferometric coherence are commonly observed across polarizations [14]. Interferometric phases derived solely from a single polarization channel frequently fail to achieve optimal quality. Incorporating multi-polarization information helps suppress decorrelation effects, improves phase estimation accuracy, and enhances the reliability of InSAR-derived deformation measurements. Current approaches for polarization optimization, while effective, often rely on complex computations and global metrics, which can limit their efficiency and applicability in large, heterogeneous mountainous regions. These challenges highlight the need for advanced InSAR frameworks capable of adaptively integrating multi-polarization data, motivating the development of the SETP-EMI method in this study.

Although polarimetric optimization has improved the interferometric phase quality of Distributed Scatterer (DS) targets to a certain extent, residual decorrelation effects may still persist. This limitation highlights the need for more advanced phase optimization strategies that can maintain accuracy under low-coherence and heterogeneous conditions [15,16,17]. Conventional strategies often rely on Sample Covariance Matrix (SCM) estimation and Statistical Homogeneous Pixel (SHP) detection, which, while effective, can be sensitive to limited observations and heterogeneous pixels [18,19,20]. Alternative approaches, such as the Total Power (TP) coherence matrix method, improve phase accuracy and efficiency compared to conventional exhaustive search methods [21,22,23,24]; however, they generally lack support for dynamic or sequential SAR processing, limiting their applicability for near-real-time monitoring. These challenges underscore the necessity of developing adaptive, sequential phase optimization frameworks—motivating the design of the SETP-EMI method presented in this study.

With the rapid growth of Synthetic Aperture Radar (SAR) archives, it becomes increasingly important to balance accurate phase optimization with efficient dynamic processing. Landslides, as environmentally sensitive geohazards, can undergo nonlinear surface deformations influenced by factors such as rainfall, water fluctuations, and seismic activity [10,13,25,26,27]. Dynamic monitoring of these changes is therefore essential for effective geohazard surveillance. To address this need, Ansari et al. [28] proposed the Sequential Estimator (SE), providing a recursive framework for dynamic InSAR processing. However, conventional SE strategies are primarily designed for single-polarized SAR and generally neglect temporal evolution of ground scattering properties, limiting their applicability in polarimetric time-series analysis.

To enhance the adaptability and dynamic processing capability of DS-InSAR in complex mountainous regions prone to landslides, this study introduces the Sequential Estimator combined with the TP polarimetric coherence matrix, employing the Expectation-Maximization Iteration (EMI) algorithm—hereafter referred to as the SETP-EMI method [29]. Conventional single-polarization and traditional polarimetric optimization methods often suffer from low coherence, phase estimation errors, and high computational costs in complex highland regions, limiting their effectiveness for precise and time-sensitive landslide monitoring. The SETP-EMI method addresses these challenges by improving phase estimation for distributed scatterers, enabling dynamic updating of scattering properties, and enhancing interferometric quality in low-coherence areas. Its application supports reliable, large-scale monitoring of potential landslide hazards and provides a robust framework for geohazard assessment and dynamic surface process analysis.

## 2. Study Area and Dataset

### 2.1. Study Area

**Figure 1:**
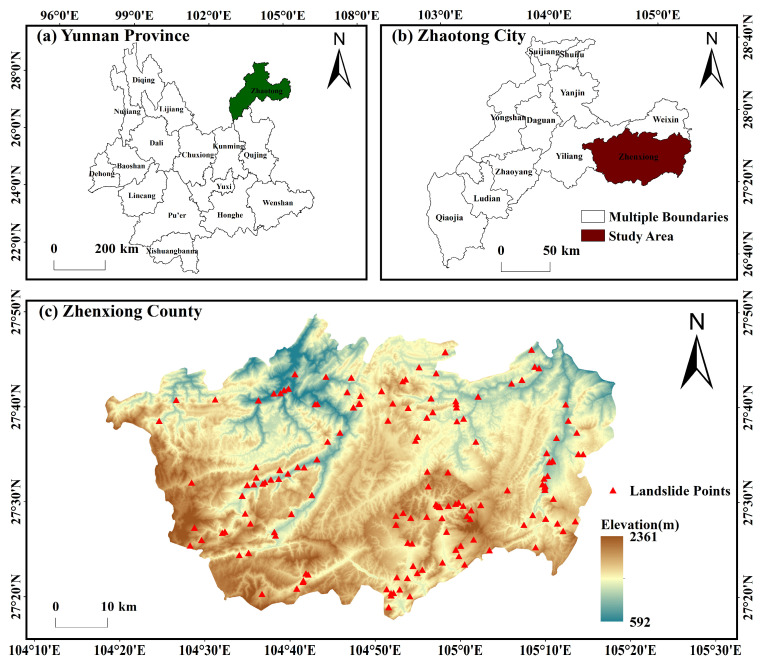
Location of the study area. (**a**) Yunnan Province with Zhaotong City highlighted; (**b**) Zhaotong City showing the study site; (**c**) DEM of Zhenxiong County with red triangles indicating landslides from 10 February 2022 to 31 January 2024.

The location of the study area is illustrated in Figure 1. Zhenxiong County, located in northeastern Yunnan at the junction of Yunnan, Guizhou, and Sichuan (104°10′–104°45′ E, 27°13′–27°45′ N), is bounded by Xuyong in Sichuan across the Chishui River to the east, Bijie and Hezhang in Guizhou to the south, Yiliang in Yunnan to the west, and Weixin County to the north [30,31]. Zhenxiong County was selected as the study area due to its location in a highland mountainous region, characterized by complex and diverse terrain, dense vegetation, and high susceptibility to landslides, making it an ideal site for evaluating InSAR-based landslide monitoring techniques. The total area of Zhenxiong County is 3696 km^2^. The landscape is a deeply dissected mountain–canyon system with mid-mountain karst; elevations increase from northeast to southwest and mainly range from 1000 to 2000 m, with dense, tiered valley networks. The climate is a subtropical plateau monsoon climate with strong altitudinal gradients, a mean annual temperature of 15 °C, frequent fog, and highly seasonal precipitation concentrated in June–August (47–76% of the annual total), culminating in 130 rainy days per year. Steep reliefs and fragile karst, combined with concentrated rainfall and anthropogenic disturbance (e.g., roads, slope excavation, mining, rural expansion), lead to frequent landslides, rockfalls, debris flows, and related geohazards that pose substantial risks to infrastructure and public safety. In addition, Zhenxiong County is located in a seismically active region, influenced by frequent moderate to strong earthquakes associated with regional fault systems, which further exacerbates slope instability and contributes to the occurrence of geohazards.

The landslides illustrated in Figure 1 are predominantly distributed across mountainous regions characterized by high elevations, steep slopes, and rugged terrain. They are mainly clustered along fault zones, deeply incised valleys, and areas with well-developed escarpments, exhibiting strong spatial aggregation and regional variability. These landslide points, provided by the Yunnan Provincial Geological Survey and verified through both remote sensing observations and field inspections, represent the source areas of the landslides. The mapped landslide points serve as ground truth for evaluating the accuracy of InSAR-based landslide detection. All landslides included in this dataset occurred within the temporal coverage of the Sentinel-1A ascending-pass SLC images used in this study, spanning from 10 February 2022 to 31 January 2024. By spatially comparing InSAR-derived deformation anomalies with these verified source locations, omission and commission errors in the interferometric results can be effectively assessed, thereby enhancing the reliability and practical applicability of InSAR techniques for landslide monitoring in complex mountainous environments.

### 2.2. Datasets

A total of 55 ascending-pass Sentinel-1A C-band Single Look Complex (SLC) images were employed in this study, covering the period from 10 February 2022 to 31 January 2024. All acquisitions were obtained in Interferometric Wide Swath (IW) mode and include dual-polarization data (VV and VH). To eliminate topographic phase contributions, the Shuttle Radar Topography Mission (SRTM) Digital Elevation Model (DEM), offering a spatial resolution of approximately 30 m and used for the DEM shown in Figure 1, served as the reference dataset for geometric correction in this study.

Following the Persistent Scatterer InSAR (PS-InSAR) image pairing strategy, the SAR scene acquired on 5 February 2023—selected based on optimal orbital stability and spatial baseline conditions—was assigned as the reference image (master). The remaining 54 acquisitions were aligned with the designated master image, after which interferometric pairs were constructed, resulting in the generation of 54 interferograms. Subsequently, a Distributed Scatterer InSAR (DS-InSAR) approach was applied for interferometric phase optimization and time-series inversion, enabling high-precision monitoring of potential landslide hazards in this complex mountainous terrain.

## 3. Methodology

**Figure 2:**
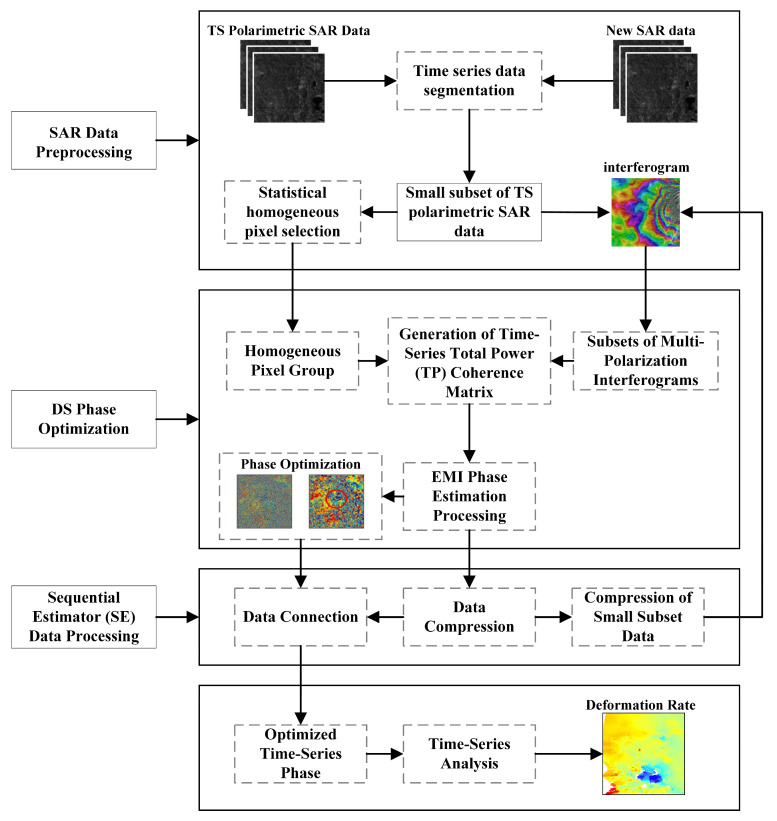
Research Framework.

The method begins by partitioning the multi-polarization SAR time-series data into subsets according to the user-specified number of acquisitions, with each subset and its compressed representation undergoing phase optimization. Differential interferograms are generated from the co-registered SLC images and their multilooked versions, and SHPs are identified to ensure spatial consistency. Multi-polarization subsets are then combined to form complex Pauli-basis scattering vectors, which, together with the SHPs, are used to compute the TP coherence matrix. The EMI estimator is applied to optimize the interferometric phase and improve coherence quality. The optimized phase is used to compress the original multi-polarization Pauli vectors, generating subset-level SLCs, and the process is iteratively applied across all subsets. Finally, EMI is employed to establish reference connections and obtain the fully optimized time-series phase. By integrating multi-polarization data into the sequential estimation framework, the SETP-EMI method balances phase estimation accuracy and computational efficiency, supports the dynamic incorporation of newly acquired SAR data without reprocessing the entire dataset, and leverages redundant polarimetric observations to enhance phase quality across the time series. The overall framework of this study is illustrated in Figure 2.

### 3.1. Time-Series InSAR Data Generation

Within polarimetric synthetic aperture radar (PolSAR), when reciprocity in backscattering is assumed, the complete polarimetric information can be expressed through the complex Pauli basis scattering vector.

(1) $$k_{Pol}=[\begin{array}{ccc} S_{HH+VV} & S_{HH-VV} & 2S_{VH} \end{array}{]}^{T}/\sqrt{2}$$

Here, $S_{HH+VV}\;$ denotes the sum of the complex-valued scattering signals acquired from the horizontal–horizontal (HH) and vertical–vertical (VV) polarization channels, while $S_{HH-VV}\;$ represents their difference. The terms $S_{HH},$ $S_{VV\;}\mathrm{and}\;S_{VH}\;$ represent the complex scattering coefficients associated with the HH, VV, and VH polarization channels, respectively. The superscript T indicates the matrix transpose operation.

In Polarimetric Interferometric SAR (PolInSAR), the polarimetric interferometric scattering vector (PolInSAR vector) $k_{PolIn}$ is composed of the master and slave complex Pauli basis scattering vectors, denoted as $k_{Pol}^{1}\;$ and $k_{Pol}^{2}\;$, respectively. The resulting six-dimensional polarimetric interferometric (PolIn) coherence matrix is defined as [32]:

(2) $$T_{PolIn}\;=\;\left\langle k_{PolIn}k_{PolIn}^{H}\right\rangle \;=[\begin{array}{cc} \left\langle T_{Pol}^{1}\right\rangle & \left\langle \Omega \right\rangle \\ \left\langle \Omega^{H}\right\rangle & \left\langle T_{Pol}^{2}\right\rangle \end{array}],$$

(3) $$k_{PolIn}=\left[\begin{array}{c} k_{Pol}^{1}\\ k_{Pol}^{2} \end{array}\right]$$

Here, H denotes the complex conjugate transpose, and ⟨·⟩  represents the multilook averaging operator. $T_{Pol}\;$ and Ω denote the polarimetric coherence matrix and the interferometric cross-correlation matrix, respectively.

For any given polarization channel ω , the complex interferometric coherence coefficient is typically expressed as [32]:

(4) $$\gamma \left(\omega \right)\;=\;\left|\gamma \omega \right|\exp\left(i\phi \left(\omega \right)\right)\;=\;\frac{\left\langle \omega^{H}\Omega \omega \right\rangle }{\sqrt{\left\langle \omega^{H}T_{Pol}^{1}\omega \rangle \langle \omega^{H}T_{Pol}^{2}\omega \right\rangle }}$$

A standard Time-Series (Polarimetric) Interferometric Synthetic Aperture Radar (TS(Pol)InSAR) dataset consists of N SAR acquisitions, from which a total of N(N − 1)/2 polarimetric interferometric pairs with varying spatial and temporal baselines can be generated. For a given polarization channel, the time-series interferometric (TSIn) coherence matrix is used to characterize the interferometric relationships among all acquisitions, and it can be estimated by averaging over a set of spatial samples [33].

(5) $$T_{TSIn}=\left\langle k_{TSIn}{\left(k_{TSIn}\right)}^{H}\right\rangle ,\;k_{TSIn}={\;\left[s^{1},\;\ldots ,\;s^{N}\right]}^{T}$$

Here, ${\;T}_{TSIn}\in \;C^{N\times N}\;$ denotes the TSIn coherence matrix, and $k_{TSIn}$ is the time-series interferometric scattering vector constructed from the N SAR acquisitions. Assuming that a resolution cell contains a large number of elementary scatterers, the complex scattering vector *k* can be modeled as a zero-mean multivariate complex Gaussian distribution, with the covariance matrix expressed as $\Sigma \;=\;E({kk}^{H})$, where E denotes the statistical expectation operator applied to ${kk}^{H}\;$.

The scattering vector *k* typically comprises two or three polarization channels, each associated with an independent TSIn coherence matrix. To reduce speckle noise and improve the phase estimation accuracy of the single-master interferograms, multilook processing can be applied. Under multi-polarization conditions, treating the TSIn coherence matrices corresponding to each polarization channel as statistical samples for joint analysis enables a more robust estimation of coherence, thereby enhancing the effectiveness of single-master phase optimization.

Based on this concept, a Time-Series Total Power (TSTP) coherence matrix ${\;T}_{TSTP}\;$ can be constructed by stacking and integrating all available polarization channels. “Total Power (TP)” refers to the sum of the squared amplitudes of the complex SAR signal across all looks or polarization channels, reflecting the overall signal strength, which is commonly used in InSAR processing to evaluate coherence, detect distributed scatterers, and perform phase optimization. Its general formulation is as follows:

(6) $$T_{TSTP}\;=\sum_{i=1}^{h}T_{TSIn\left(Pol_{i}\right)}=\sum_{i=1}^{h}\left\langle k_{TSIn\left(Pol_{i}\right)}k_{TSIn\left(Pol_{i}\right)}^{H}\right\rangle$$

For fully polarimetric data, the polarimetric vector $\mathrm{Pol}\;\in \;\left\{\sqrt{2}\left(S_{\mathrm{HH}}\;+\;S_{\mathrm{VV}}\right),\;\sqrt{2}\left(S_{\mathrm{HH}}-S_{\mathrm{VV}}\right),\;2\sqrt{2}S_{\mathrm{VH}}\right\}/2$. For cross-polarized data, the polarimetric vector is defined as $\mathrm{Pol}\in \left\{S_{\mathrm{xx}},\;2S_{\mathrm{VH}}\right\}\;.\;$ Here, h  denotes the number of polarization channels used. In this study, a Pauli-basis polarization TSIn coherence matrix is expressed as ${\;T}_{TSIn}\in \;C^{N\times N}$.

### 3.2. Sequential EMI with Multi-Polarization SAR

Phase Linking (PL) theory is regarded as an effective approach for reconstructing interferometric phase and is typically implemented using multilooked TSIn coherence matrices. In this study, the TSIn coherence matrix is replaced by the TSTP coherence matrix for improved performance [29].

Under the complex Wishart statistical model framework, the maximum likelihood estimation (MLE) of the single-master (SM) interferometric phase vector $\theta \;={\;\left[\theta_{1},{\;\theta }_{2},\;\ldots ,{\;\theta }_{N}\right]}^{T}\;$ can be obtained as follows [33]:

(7) $$\theta_{MLE}\;=\;argmax_{\theta }\left\{\Theta^{H}\left({\left|\gamma \right|}^{-1\circ }T_{TSTP}\right)\Theta \right\}$$

The operator ∘  denotes the Hadamard product (element-wise multiplication), and Θ = exp(jθ) . The matrix γ  represents the true coherence matrix. In practical applications, since γ is unknown, its magnitude |γ|  is typically approximated using the absolute value of the empirical TSTP coherence matrix $T_{TSTP}\;$.

Although the Phase Linking (PL) method provides high-accuracy solutions to the MLE problem, it is computationally intensive. To address this issue, Ansari et al. [34] proposed the Expectation-Maximization Iteration (EMI) algorithm, which offers an efficient solution to the MLE formulation. The EMI method performs eigenvalue decomposition (EVD) of the coherence matrix and selects the eigenvector corresponding to the smallest eigenvalue as the phase estimate:

(8) $$\theta_{EMI}\;=\;argmin_{\theta }\left\{u^{H}\left({\left|T_{TSTP}\right|}^{-1\circ }T_{TSTP}\right)u\right\}$$

Here, u denotes the eigenvector corresponding to the smallest eigenvalue of the matrix ${\;\left|T_{TSTP}\right|}^{-1\circ }T_{TSTP}\;$, where ∘  represents the Hadamard (element-wise) product. The estimated single-master (SM) interferometric phase vector $\theta_{EMI}\;$ is then obtained by taking the complex argument of u. $e\theta_{EMI}\;=\;∠u\;$ denotes the phase angle of a complex number.

However, this method does not avoid redundant computations on previously processed data, resulting in substantial computational overheads. To address this issue, Ansari, De Zan and Bamler [28] proposed a Sequential Estimator designed for the efficient processing of large-scale InSAR datasets. Compared with conventional sequential estimation approaches, the method employed in this study incorporates two key improvements: continuous updating of the identified Statistical Homogeneous Pixel (SHP) set using the sequential estimation framework, and compression of SAR data across different polarization channels [29].

Assume that a large-scale dataset consists of n SLC SAR images and h polarization channels. This dataset is partitioned into τ subsets. Each of the first τ-1 subsets contains ξ images, while the last subset may contain a different number of acquisitions. Within each subset, l homogeneous pixels are identified along statistically homogeneous paths.

To reduce the dimensionality of the original data, the high-dimensional dataset ${\tilde{Z}}^{\xi •l•h}\;$ is projected into a low-rank subspace ${\tilde{Z}}^{m•l•h}\;$ using a transformation basis $T\;=\;\nu_{1};\;\ldots ;\;\nu_{m}\;$, where m denotes the target dimensionality after compression.

In scenarios where DS exhibit multiple dominant displacement behaviors along the elevation axis within the SAR resolution cell, a higher signal subspace dimension m  is typically required to preserve the structural information of the deformation signals [28]. However, under the assumption of a single dominant scattering mechanism, the dimensionality can be simplified to m = 1.

Unlike the compression process for single-polarization data, in the case of multi-polarization SAR, each polarization channel must be compressed independently. For fully polarimetric SAR data, three low-rank compressed subspaces are generated separately for the three Pauli scattering components (h = 3). The normalized maximum likelihood (ML) signal vector ${\;\nu }_{ML}\;=\;\frac{exp\left(j•\theta_{EMI}\right)}{∥exp\left(j•\theta_{EMI}\right)∥}$ is used to replace the first basis vector $\nu_{1}\;$ in the original transformation basis, thereby constructing an updated basis matrix $T\;=\;\{\nu_{ML};{\;\nu }_{1};\;\cdot \cdot \cdot ;{\;\nu }_{m-1}\}$. When compressing the SAR data of the first subset, a linear transformation is applied to project the data onto the subspace defined by the updated basis matrix T.

(9) $${\tilde{Z}}_{1}^{1•l•h}={\;T}^{H}Z_{1}^{\xi •l•h}$$

When processing the second subset, the compressed data from the first subset ${\tilde{Z}}_{1}$ is combined with the raw data of the second subset $Z_{2}$ for joint analysis, and the optimized phase values are obtained using MLE. This procedure is iteratively applied to each subsequent  τ-th subset (τ > 1), where each subset is jointly processed with the compressed result from the previous subset to achieve increasingly accurate phase estimates.

Specifically, the processing strategy for the τ-th subset is as follows:

(10) $${\hat{Z}}_{\tau }\;=\;\left\{{\tilde{Z}}_{1};{\tilde{Z}}_{2};\;\ldots ;{\;Z}_{\tau }\right\}$$

Here, ${\hat{Z}}_{\tau }$ denotes the new dataset to be optimized in the current iteration, where $Z_{\tau }$ represents the raw data of the τ-th  subset, and ${\tilde{Z}}_{i}$ denotes the compressed data from the *i*-th subset (i = 1, ..., τ–1). In the τ-th sequence, the EMI method is employed to estimate the interferometric phase of each sequence with respect to its designated reference image.

The compressed SLC data from each subset is then structured into a new subset of the TSTP matrix, denoted as $T_{TSTP\_sub}$. Within this framework, the EMI algorithm assigns a unique arbitrary reference acquisition to each subset, ensuring phase consistency across the entire time series.

(11) $${\hat{\theta }}_{cal\;}=\;{\mathrm{argmin}}_{u}\left\{u^{H}\left({\left|T_{TSTP\_sub}\right|}^{-1\circ }T_{TSTP\_sub}\right)u\right\}$$

The corrected interferometric phase of the *i*-th sequence is expressed as:

(12) $${\hat{\theta }}_{unified}\;=\;{\hat{\theta }}^{i}\;+\;{\hat{\theta }}_{cal}\left(i\right)$$

Here, ${\hat{\theta }}^{i}\;$ denotes the maximum likelihood estimated phase of the *i*-th sequence, ${\hat{\theta }}_{cal}\left(i\right)\;$ represents the reference-calibrated phase, and ${\hat{\theta }}_{unified}$ is the final unified phase for the *i*-th sequence. Through this strategy, newly acquired SAR data are sequentially partitioned, recursively estimated, and progressively linked, ultimately yielding an optimized interferometric phase result for the complete time series.

The SETP-EMI method introduces a series of multidimensional advancements over traditional polarimetric InSAR frameworks, enhancing both processing efficiency and phase estimation accuracy for complex mountainous environments. Specifically, it integrates multi-polarization observations to construct a TP coherence matrix, thereby improving the sensitivity of interferometric phase estimation to complex scattering mechanisms and enhancing phase stability in low-coherence regions. Additionally, the incorporation of a Sequential Estimator enables recursive processing and dynamic updating of newly acquired SAR acquisitions, which significantly reduces computational and storage demands while supporting timely and scalable InSAR analysis. Furthermore, the use of the EMI algorithm facilitates MLE of interferometric phase, increasing the robustness and precision of phase history reconstruction in heterogeneous terrain. By simultaneously addressing the challenges of data volume, temporal variability, and scattering complexity, the SETP-EMI method offers a technically effective and operationally efficient pathway for high-precision multi-polarization InSAR deformation monitoring in geologically active mountainous regions.

To enhance the reproducibility and methodological transparency of the proposed SETP-EMI approach, the key data processing steps and core parameter configurations are summarized in Table 1. The method integrates multi-polarization information, a sequential estimator, and a maximum likelihood-based phase optimization algorithm. In practical implementation, the configuration of parameters exerts a decisive influence on the accuracy, efficiency, and robustness of interferometric outcomes.

## 4. Results

### 4.1. Parameter Settings

**Figure 3:**
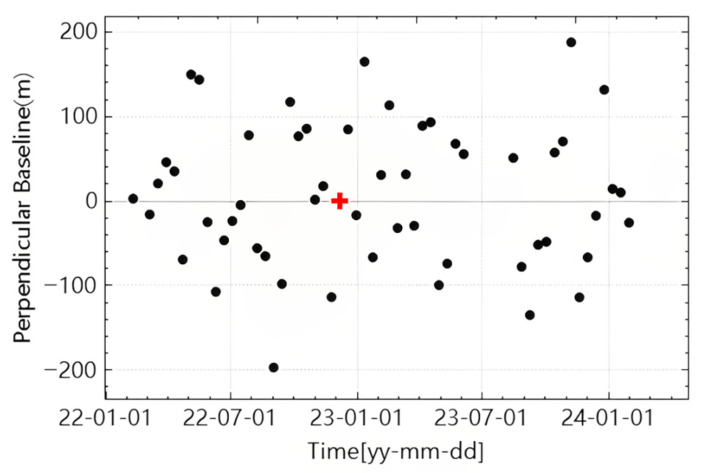
Baseline configuration of the datasets used in PS-InSAR and DS-InSAR processing. The red cross indicates the selected master image.

**Figure 4:**
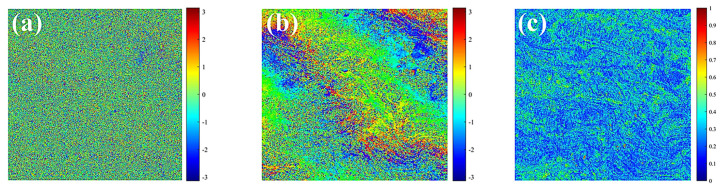
Full-scene interferograms between Sentinel-1 acquisitions. (**a**) Original unfiltered interferogram; (**b**) Interferogram after homogeneous temporal filtering; (**c**) Map of extracted statistically homogeneous pixels.

To generate accurate and high-resolution landslide deformation monitoring maps for Zhenxiong County, Zhaotong City, a polarimetric phase optimization approach combining Expectation-Maximization Iteration (EMI) and a Sequential Estimator (SE) was employed and applied to ascending-pass Sentinel-1 SAR data acquired between 2022 and 2024. To verify result reliability, InSAR-derived deformation estimates were cross-validated with displacement records from fixed ground monitoring stations within the study area. In the subsequent experiments, the Sentinel-1 PolSAR SLC acquired on 7 December 2022 was designated as the master image, and the remaining acquisitions were used as slave images to form all possible interferometric pairs. Table 2 summarizes the key imaging parameters of the Sentinel-1A time-series dataset used in this study. The corresponding perpendicular and temporal baselines are illustrated in Figure 3. Topographic phase simulation was carried out using the Shuttle Radar Topography Mission (SRTM) Digital Elevation Model with a spatial resolution of 30 m. For the TS-PolInSAR data that had undergone homogeneous temporal filtering, a local 7 × 7 window was applied, and the Expectation-Maximization Iteration (EMI) method was used to estimate the ESM (Estimated Single-Master) phase. Figure 4 shows examples of interferograms and homogeneous pixel extraction during Sentinel-1 data processing. This figure illustrates two interferograms and a homogeneous pixel map generated during the processing workflow. Figure 4a shows the original interferogram without filtering, which exhibits significant phase noise due to the long temporal baseline. Figure 4b presents the interferogram after homogeneous temporal filtering, where the fringe patterns are partially suppressed but some residual noise remains. Figure 4c displays the map of extracted statistically homogeneous pixels, which effectively enhances phase consistency and retains good interferometric quality, particularly in mountainous areas.

### 4.2. Landslide Detection and Representative Case Studies

#### 4.2.1. Landslide Locations and Optical Imagery Overview

After completing the full processing workflow, including data preprocessing, interferogram generation, phase optimization using SETP-EMI, and time-series inversion for both PS and DS points, the total computational time required for the 55 Sentinel-1A acquisitions over Zhenxiong County was approximately 3.5 h on a workstation equipped with a 16-core CPU and 32 GB RAM.

The LOS (Line-of-Sight) surface deformation results of the study area were derived from ascending Sentinel-1 data using the SETP-EMI method. Overall, the surface deformation exhibits pronounced spatial heterogeneity, with distinct deformation anomalies observed in localized areas. The annual average LOS deformation rates along the ascending orbit range from −87 to 37 mm/a, and in some areas the absolute deformation rate exceeds 20 mm/a, indicating strong surface activity and suggesting a high potential for landslide development.

Based on the above surface deformation results, and in combination with previous studies on InSAR-based landslide monitoring as well as engineering experience, this study adopts the absolute value of the annual average deformation rate as the indicator for identifying potential landslide deformation zones. Areas with an absolute deformation rate greater than 15 mm/a are classified as suspected landslide-active zones. Furthermore, the InSAR-derived deformation anomalies are integrated with high-resolution optical imagery for visual interpretation, enabling the verification of geomorphological features indicative of landslide activity, such as scarps, tension cracks, and disturbed surface textures. This joint analysis maintains high sensitivity to slow landslide deformation processes while effectively reducing the influence of noise, orbital residuals, and localized non-landslide-related deformation, making it suitable for preliminary landslide screening under the complex mountainous terrain conditions of the study area.

**Figure 5:**
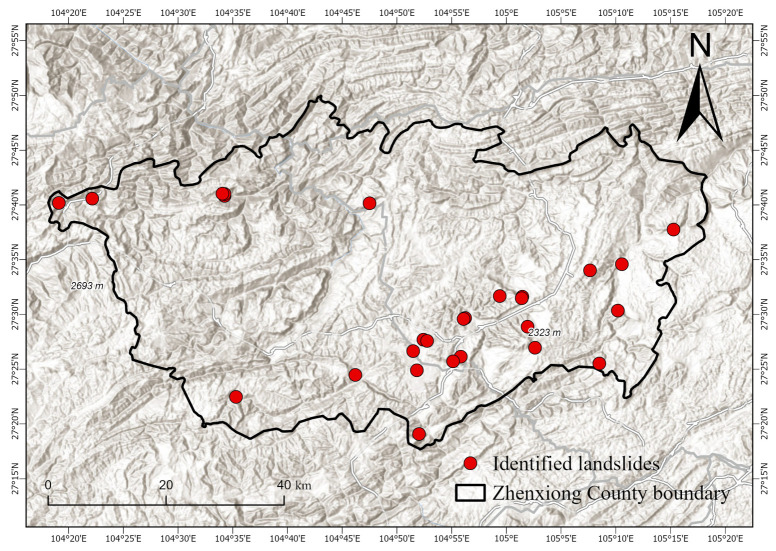
Spatial distribution of suspected landslide deformation areas identified from ascending-orbit InSAR results.

**Figure 6:**
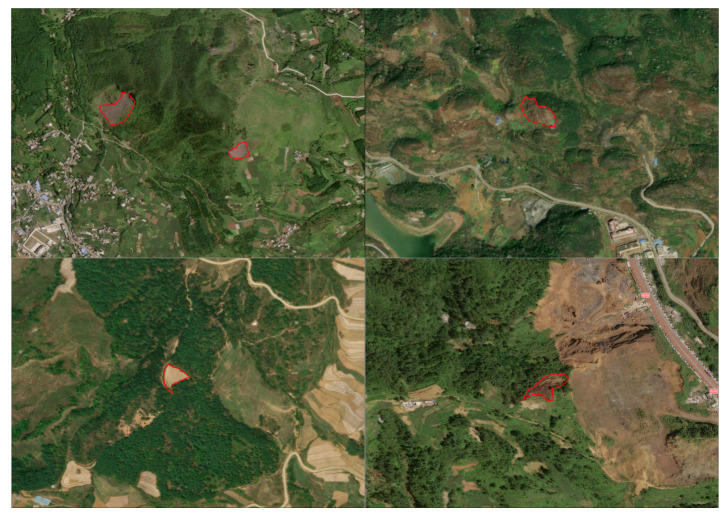
High-resolution optical images of four representative suspected landslide areas for validation of InSAR detection results (The red frames indicate the boundaries of the landslides).

Based on the above criteria, a total of 28 suspected landslide deformation areas were identified from the ascending-orbit InSAR deformation results. Figure 5 illustrates the spatial distribution of these suspected landslide deformation areas within the study region. In terms of their spatial characteristics, these areas are predominantly located in mountainous zones with steep slopes and pronounced topographic relief, showing good consistency with the geomorphological and geological settings of landslide-prone areas in the study region. To further verify the reliability and credibility of the InSAR-based identification results, several representative suspected landslide areas were selected for comparative analysis using high-resolution optical imagery. Figure 6 presents the optical image characteristics of five typical suspected landslide areas, in which landslide morphology, landslide boundaries, and evident surface disturbance features can be clearly recognized. These observations provide intuitive and reliable evidence for subsequent analyses of landslide activity based on InSAR time-series deformation characteristics.

#### 4.2.2. InSAR-Detected Deformation and Time-Series Analysis

To further investigate landslide activity at the slope scale and its temporal evolution, two representative suspected landslide deformation areas were selected from the InSAR-detected results for detailed analysis. These two areas exhibit clear deformation signals and dense InSAR point coverage, and their spatial patterns are consistent with geomorphic features interpreted from high-resolution optical imagery, making them suitable examples for demonstrating the capabilities of the proposed SETP-EMI method.

**Figure 7:**
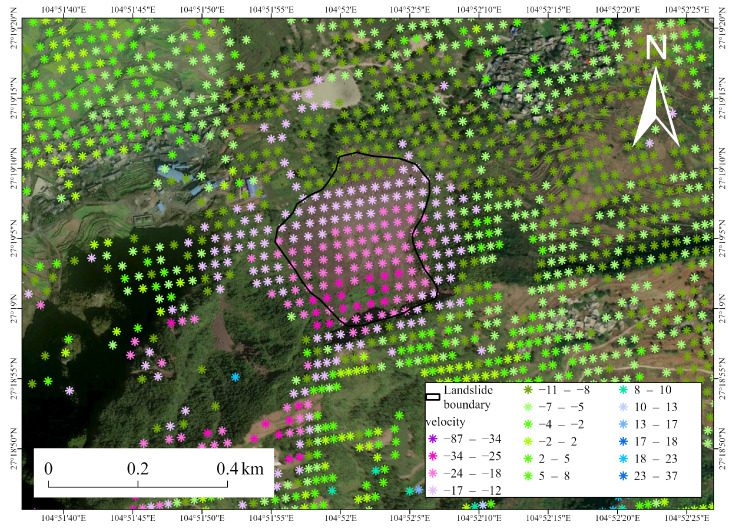
Spatial distribution of InSAR monitoring points in Landslide Site 1.

**Figure 8:**
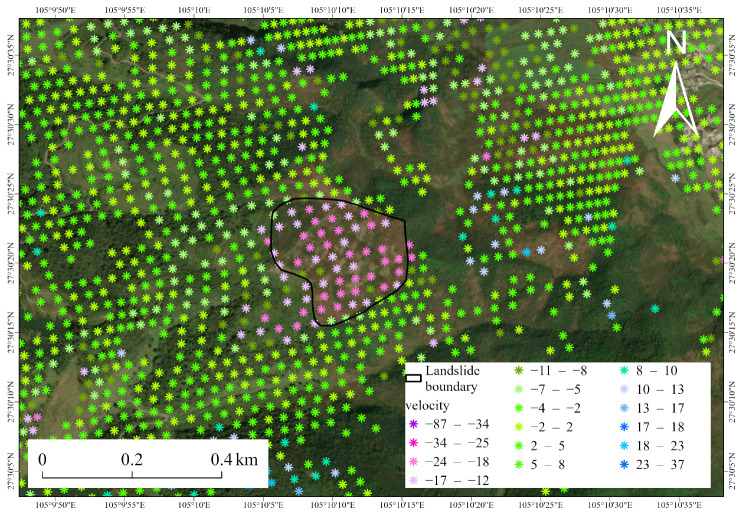
Spatial distribution of InSAR monitoring points in Landslide Site 2.

Figure 7 and Figure 8 illustrate the spatial distribution of InSAR monitoring points across the two typical landslide areas. The points are primarily concentrated within the inferred landslide boundaries, showing continuous and coherent deformation patterns. This indicates that the detected signals reflect genuine slope-scale surface movement rather than isolated noise. The high density and spatial continuity of InSAR points within the landslide bodies highlight the effectiveness of the SETP-EMI approach in preserving distributed scatterers in complex mountainous terrain.

**Figure 9:**
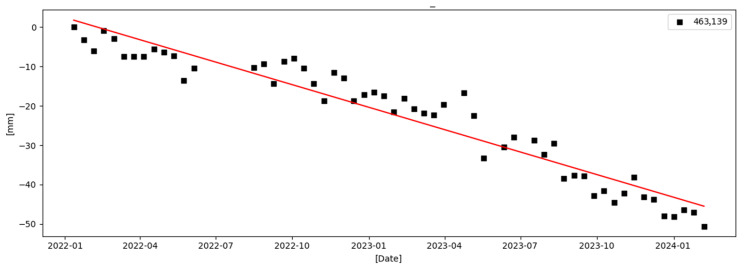
Cumulative LOS deformation time series for Landslide Site 1.

**Figure 10:**
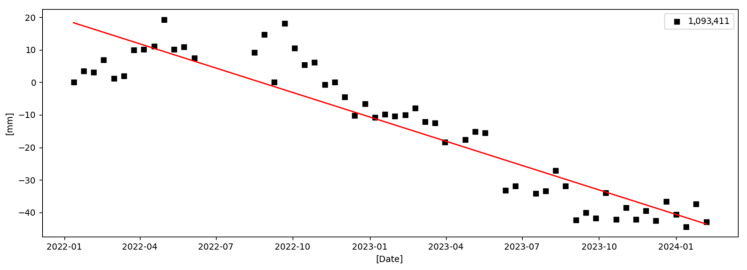
Cumulative LOS deformation time series for Landslide Site 2.

Based on these distributions, representative InSAR points were selected from each landslide, and their Line-of-Sight (LOS) cumulative deformation time series were extracted. Figure 9 and Figure 10 present the corresponding time-series deformation curves. Both landslides display progressive and sustained deformation trends over the monitoring period, consistent with the patterns observed in the annual average deformation rate maps. Such gradual deformation is characteristic of slow-moving or creeping landslides.

The time-series curves show no abrupt jumps or phase discontinuities, indicating that the landslides are undergoing stable yet continuous activity rather than sudden catastrophic failure. This demonstrates the advantage of time-series InSAR in capturing slow-moving slope deformation processes, which are difficult to detect using conventional ground-based monitoring methods over large mountainous areas.

Overall, the spatial distribution of InSAR points and the corresponding temporal deformation evolution for these representative landslides validate the reliability and credibility of the InSAR results. These findings indicate that the SETP-EMI-based time-series InSAR approach can not only identify potential landslide deformation areas at the regional scale but also provide physically meaningful deformation measurements at the slope scale, offering essential technical support for long-term landslide monitoring and early warning in complex mountainous terrain.

### 4.3. Comparative Analysis of Different InSAR Methods

This study employed three InSAR techniques for monitoring surface deformation: PS-InSAR, SBAS-InSAR, and DS-InSAR using the proposed SETP-EMI method. In SBAS-InSAR processing, a temporal baseline threshold of 180 days and a coherence threshold of 0.2 were set [35]. During the preprocessing stage, the Sentinel-1 data were processed using ESA’s SNAP toolbox, including radiometric calibration, co-registration, interferogram generation, removal of topographic phase, and phase unwrapping.

For PS-InSAR and DS-InSAR alike, the preprocessed data and interferometric phases optimized using the SETP-EMI method were imported into the StaMPS platform to perform time-series deformation analysis for Persistent Scatterer (PS) points and Distributed Scatterer (DS) points, respectively. The core strength of the SETP-EMI-based DS-InSAR approach lies in its ability to jointly model and compress multi-temporal interferometric phases in both space and time, thereby enabling the extraction of high-density, stable DS deformation measurements even in low-coherence regions.

To reduce atmospheric disturbances, all three approaches applied atmospheric delay corrections through the Generic Atmospheric Correction Online Service (GACOS), which further improved the accuracy and spatiotemporal consistency of the deformation retrievals. By fully leveraging the advantages in spatial coverage and signal-to-noise ratio, these methods produced high-precision and high-density surface deformation fields.

**Figure 11:**
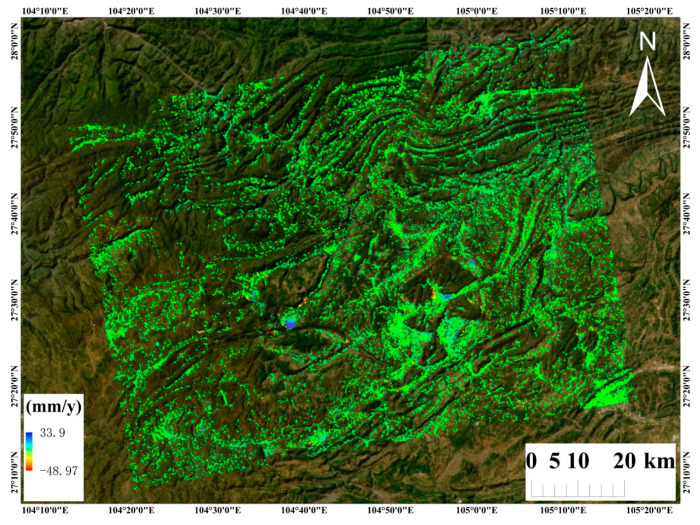
Surface deformation velocity map derived from 55 ascending-pass Sentinel-1 acquisitions using PS-InSAR.

**Figure 12:**
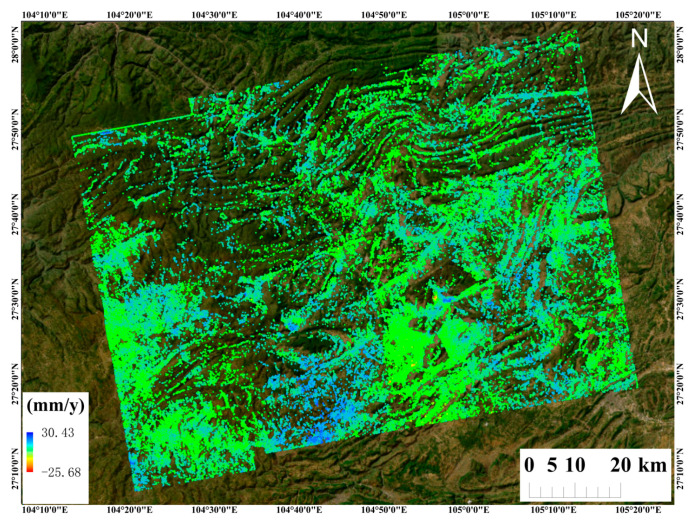
Surface deformation velocity map derived from 54 ascending-pass Sentinel-1 acquisitions using SBAS-InSAR.

**Figure 13:**
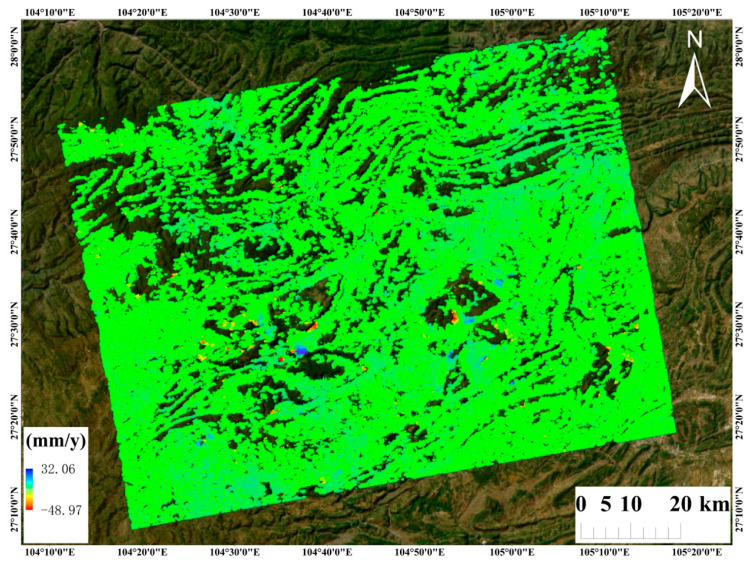
Surface deformation velocity map derived from 55 ascending-pass Sentinel-1 acquisitions using DS-InSAR with interferometric phase optimization based on the SETP-EMI method.

Figure 11 presents the PS-InSAR results derived from 55 ascending-track Sentinel-1 images; Figure 12 shows the SBAS InSAR results using 54 images; and Figure 13 shows the time-series deformation results derived from the DS-InSAR approach based on SETP-EMI. Evidently, the deformation regions identified by the three methods show substantial overlap. Notably, the DS-InSAR method based on SETP-EMI maintains considerable mon-itoring capability even after image compression, significantly enhancing processing efficiency.

**Figure 14:**
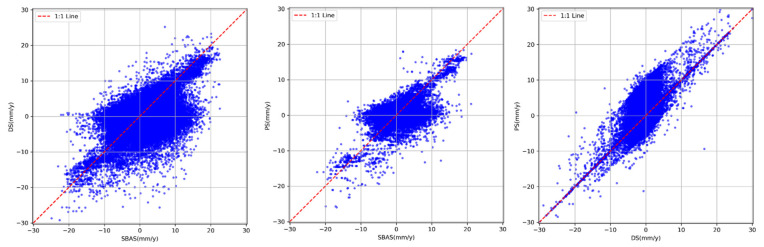
Scatter plots of deformation rates derived from different InSAR methods.

In order to evaluate the agreement among the three InSAR approaches—PS-InSAR, SBAS-InSAR, and DS-InSAR enhanced by the SETP-EMI method—a point-by-point correlation analysis was carried out with ascending-pass Sentinel-1 acquisitions. Considering potential discrepancies in the geometric positioning of measurement points (MPs) due to differences in imaging geometry, all MPs were projected onto a unified coordinate system. The vertical deformation rate within each grid cell was computed as the average of the deformation rates of MPs located within that cell. Statistical metrics including the Pearson correlation coefficient (R), mean error (ME), and standard deviation (STD) were used to quantitatively assess the differences between methods, as shown in Figure 14. The results indicate that PS-InSAR and SETP-EMI-based DS-InSAR exhibit excellent agreement, with an R value of 0.9892, a mean error of –3.87 mm/year, and a standard deviation of 2.15 mm/year, suggesting highly consistent deformation trends and magnitudes. By contrast, the consistency between SBAS-InSAR and PS-InSAR is relatively low, with a correlation coefficient of 0.7935, a mean error of –6.58 mm/year, and a standard deviation of 6.72 mm/year. When compared with DS-InSAR optimized through SETP-EMI, the correlation increases to 0.8764, accompanied by a mean error of –5.04 mm/year and a standard deviation of 5.29 mm/year, though residual local discrepancies in SBAS-derived deformation remain evident. Overall, the SETP-EMI method demonstrates strong practical utility and accuracy, achieving high deformation coherence with PS-InSAR while significantly enhancing coherent point density and spatial continuity—making it particularly suitable for large-scale landslide monitoring in complex mountainous regions.

The number and spatial distribution density of measurement points (MPs) extracted by the three InSAR techniques are summarized in Table 3. The comparative analysis reveals significant differences in MP density and spatial coverage among the methods. The PS-InSAR approach identified 110,653 persistent scatterers; however, due to its reliance on highly coherent and spatially discrete targets, its coverage is limited in non-urban and low-coherence areas. The SBAS-InSAR method extracted 779,367 MPs, showing improved spatial density compared to PS-InSAR and better suitability for monitoring over broader, relatively stable regions. In contrast, the DS-InSAR technique optimized using the proposed SETP-EMI method identified 2,489,144 distributed scatterers, representing an approximate 22.5-fold and 3.2-fold increase compared with the PS-InSAR and SBAS-InSAR results, respectively. The DS points derived from SETP-EMI exhibit significantly enhanced spatial density and a more uniform distribution, particularly in areas with high topographic complexity and vegetation cover, where traditional methods typically underperform. Overall, the results demonstrate that under consistent processing parameters, the SETP-EMI method—through temporal phase compression and denoising—substantially improves the signal-to-noise ratio and effective MP extraction. This leads to higher data utilization efficiency and broader spatiotemporal coverage, making the method especially suitable for fine-scale landslide monitoring and risk assessment in mountainous and landslide-prone regions.

For a comprehensive understanding of landslide hazard distribution within the study region, a dataset of 152 verified landslide locations was assembled from field survey records provided by the Yunnan Provincial Geological Survey. These landslide points are shown as red circles in Figure 1. Following the extraction of interferometric deformation results, landslide detection and delineation were performed using a threshold-based classification method combined with spatial clustering analysis. Within the study area, potential landslide locations were identified separately using PS-InSAR, SBAS-InSAR, and DS-InSAR enhanced by the SETP-EMI framework, and the results were compared against field-surveyed landslide points. The detection results indicate that 103 landslides were identified using PS-InSAR, 117 using SBAS-InSAR, and 143 using the SETP-EMI approach. Compared to the other methods, SETP-EMI demonstrated superior performance in terms of point density, deformation continuity, and noise suppression. The spatial distribution of the landslide points extracted using SETP-EMI showed a high level of agreement with the field investigation data. The landslide detection rate (hit rate) achieved by the SETP-EMI method was 94.1%, significantly outperforming SBAS (77.0%) and PS-InSAR (67.8%). In particular, SETP-EMI maintained stable detection performance in areas with steep slopes and dense vegetation cover, confirming its adaptability and robustness under complex mountainous terrain conditions. The spatial resolution of the InSAR monitoring points ranges from approximately 20 m in dense areas to around 50 m in sparse areas, enabling effective identification of landslide bodies and their local deformation features, thus providing a reliable basis for early warning and fine-scale landslide monitoring.

Despite achieving a 94.1% detection rate, some landslides were still missed. The primary causes include severe phase decorrelation caused by dense vegetation, rapid surface changes, or intense rainfall, which reduces the coherence of the interferometric phase, and geometric distortions in localized areas, particularly on steep slopes where radar shadowing or layover occurs, preventing accurate measurement of surface displacement. Additionally, small-scale landslides with low deformation magnitudes may produce signals below the detection threshold. These limitations highlight the importance of integrating high-resolution optical imagery, geological maps, and other multi-source datasets in future studies to enhance the completeness, accuracy, and reliability of landslide detection in complex mountainous terrain.

### 4.4. Comparison of Sentinel-1 InSAR and Leveling-Derived Deformation

**Figure 15:**
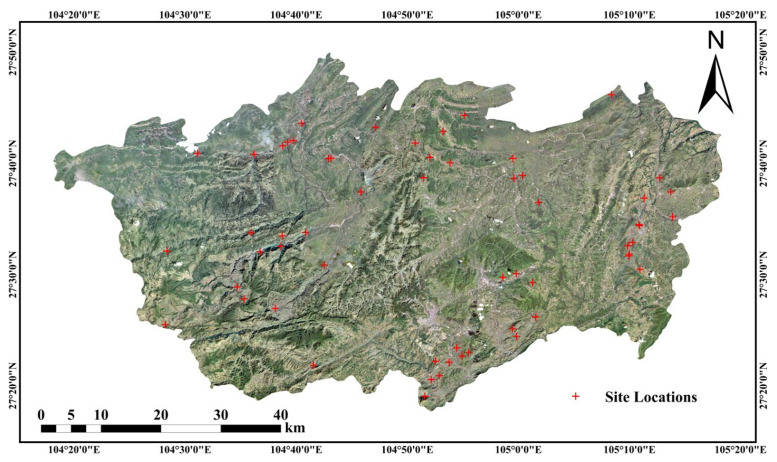
Distribution of ground-based vertical deformation measurement stations. The figure shows the spatial locations of monitoring stations used for validating InSAR-derived vertical deformation results within the study area.

**Figure 16:**
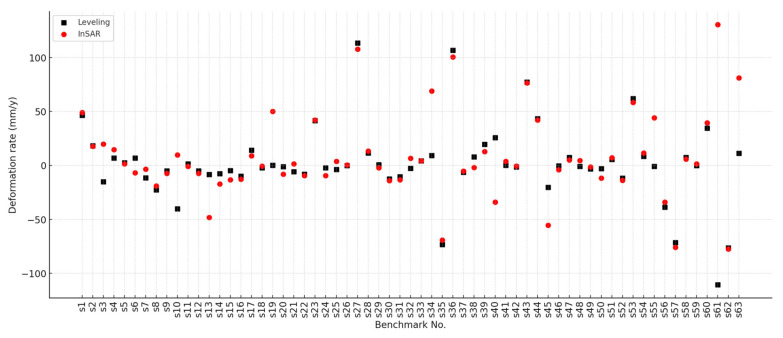
Comparison between InSAR-derived deformation results (2022–2024) and leveling measurements.

A comparative analysis was conducted between the InSAR-derived surface deformation results based on the Sentinel-1 dataset and leveling measurements from 64 stations collected by the Yunnan Provincial Geological Survey during the period from 2022 to 2024. The two datasets share a two-year overlap in temporal coverage, and the spatial distribution of the leveling stations is illustrated in Figure 15. To enable a meaningful comparison, InSAR measurement points within a 100 m × 100 m window centered on each leveling station were extracted, and their annual average deformation rates over the overlapping period were calculated. The results of the comparison are illustrated in Figure 16. A standard deviation of 5.21 mm/year was observed between the annual average deformation rates obtained from InSAR analysis and ground-based leveling measurements, indicating overall good agreement. Minor discrepancies between the two datasets may be attributed to spatial mismatches between InSAR and leveling points, as well as slight temporal differences in data acquisition, which could lead to deformation offsets.

Overall, the Sentinel-1-derived results show a high level of consistency with ground-based leveling measurements, validating the effectiveness and reliability of the proposed algorithm for surface deformation monitoring. These findings suggest strong potential for large-scale application in highland and mountainous regions.

## 5. Discussion

### 5.1. Method Applicability Analysis

In this work, the SETP-EMI method, which combines polarimetric information with a sequential estimation strategy, was employed to improve the accuracy and robustness of DS-InSAR for landslide monitoring in complex terrain. Experimental results demonstrate that DS-InSAR outperforms both PS-InSAR and SBAS-InSAR in non-persistent scatterer regions. Taking the mountainous and hilly terrain of Zhenxiong County as a representative example, the traditional PS-InSAR method suffers from spatial gaps due to the sparse distribution of highly coherent scatterers. Although the SBAS-InSAR approach offers improved spatial coverage, it remains susceptible to phase noise and spatial inconsistencies in low-coherence areas, often resulting in localized deformation misinterpretations. In contrast, the SETP-EMI-optimized DS-InSAR method, by integrating VV/VH dual-polarization channels and constructing a total power coherence matrix, significantly enhances phase stability and coherent point density in low-coherence environments such as farmlands, shrublands, and terraced fields. This leads to improved spatial continuity of the deformation field and effectively addresses the coverage limitations associated with conventional InSAR techniques.

Comparative analysis with ground-based vertical displacement measurements further confirms that the SETP-EMI method achieves high deformation estimation accuracy while maintaining a high density of measurement points. Each InSAR-derived deformation point was spatially matched with the nearest leveling station within a 50 m radius. The comparison indicates a standard deviation of 5.21 mm/year in annual vertical deformation rates, demonstrating the high accuracy and robustness of the method across different terrain types, including steep slopes, terraces, and densely vegetated areas. Some deviations are mainly caused by severe phase decorrelation, geometric distortions in localized areas (e.g., radar shadowing or layover on steep slopes), and small-scale landslides with low deformation magnitudes below the detection threshold. These results underscore the reliability of the SETP-EMI approach for operational landslide monitoring and its suitability for integration into early-warning systems.

### 5.2. Data Volume and Processing Efficiency

Within the framework of time-series InSAR analysis, the increasing number of SAR acquisitions over extended observation periods poses significant challenges in terms of computation and storage. This issue becomes even more prominent in the context of multi-polarization and long-term observations, where data redundancy can severely impact processing efficiency. In response to these challenges, a Subset-based Estimation with Temporal Partitioning (SETP) approach is adopted in this study, which achieves efficient data compression and recursive optimization while maintaining the overall quality of phase estimation.

Through the subdivision of the SAR time series into multiple temporal segments, the EMI algorithm is employed within each subset to perform, the method avoids redundant re-computation of phase estimates from previously processed images. This significantly reduces memory consumption and computational load. After completing the full processing workflow—including data preprocessing, interferogram generation, SETP-EMI-based phase optimization, and time-series inversion for both PS and DS points—the total computational time required for the 55 Sentinel-1A acquisitions over Zhenxiong County was approximately 3.5 h on a workstation equipped with a 16-core CPU and 64 GB RAM. This demonstrates that the method not only provides high-accuracy deformation measurements but also supports near-real-time updates, which is critical for rapid geohazard assessment and early warning.

More importantly, the SETP approach supports dynamic updates for newly acquired SAR scenes. Based on previously estimated results, the algorithm can integrate new acquisitions recursively without reprocessing the entire dataset. This feature is particularly valuable for monitoring geohazards such as landslides that require continuous observation over time. It is especially well-suited for high-revisit SAR missions such as Sentinel-1 or upcoming polarimetric SAR constellations, laying the foundation for near-real-time deformation monitoring.

### 5.3. Limitations and Uncertainty

Despite the demonstrated effectiveness of the SETP-EMI approach in the present study, several limitations remain. While the method performs robustly in the low-to-mid elevation terrain of Zhenxiong County, its application in high-altitude mountainous regions may encounter considerable challenges. Seasonal snow cover, freeze–thaw cycles, and harsh environmental conditions can significantly reduce coherence, potentially decreasing both the density and accuracy of DS-InSAR measurements. These factors must be carefully considered when extending the method to such environments, as they can impede reliable deformation extraction and complicate landslide monitoring efforts.

Second, the performance of polarimetric optimization and sequential estimation remains sensitive to initial parameter settings and homogeneous pixel identification. In regions with extreme topography or severe phase decorrelation, reliable phase estimation remains challenging. Moreover, the current SETP-EMI framework focuses primarily on detecting linear deformation trends and may have limited capacity in responding to abrupt or nonlinear landslide events, which warrants further investigation.

Future research should focus on the following aspects for continued improvement and expansion: (1) incorporating artificial intelligence (AI) and deep learning techniques for automated identification and classification of landslide deformation patterns, thereby enhancing the interpretability of results; (2) integrating high-resolution polarimetric SAR data (e.g., GF-3, TerraSAR-X) with multi-source optical and LiDAR datasets to develop a multi-modal landslide detection framework; (3) improving polarimetric feature selection and adaptive channel weighting mechanisms to achieve more robust and data-driven optimization of polarization configurations; and (4) coupling InSAR-derived deformation with meteorological, hydrological, and geological indicators to establish a “deformation–trigger–response” framework for landslide early warning and prediction, thereby extending the applicability of time-series InSAR toward proactive disaster risk management.

## 6. Conclusions

The proposed Sequential Estimation and Total Power-Enhanced Expectation–Maximization Inversion (SETP-EMI) approach exhibits marked advantages for landslide monitoring in complex mountainous terrain. Applied to 55 Sentinel-1A dual-polarization acquisitions (2022–2024) over Zhenxiong County, Yunnan Province, the method retrieved approximately 2.49 million distributed scatterer (DS) points—representing approximately 22.5-fold and 3.2-fold increases relative to PS-InSAR and SBAS-InSAR, respectively—and attained a landslide detection rate of 94.1%. Validation against 64 leveling stations indicated a standard deviation of 5.21 mm/year in annual deformation rates, confirming its high accuracy and robustness. By synergistically integrating multi-polarization observations, a sequential estimation strategy, and EMI-based phase optimization, SETP-EMI markedly improves phase stability and spatial coverage in low-coherence environments, while enabling near-real-time dynamic updates. These capabilities make it well suited for large-scale geohazard surveillance. Future efforts will focus on leveraging high-resolution SAR, multi-source data fusion, and artificial intelligence techniques to further mitigate decorrelation effects and enhance automation. The results and the SETP-EMI model are expected to benefit local geohazard monitoring agencies, infrastructure management authorities, and research institutions, providing reliable data for early warning and risk mitigation.

## Figures and Tables

**Table 1:** Key processing parameter configurations of the SETP-EMI method.

Parameter Category	Parameter Name	Value or Description	Notes
Data Type	Polarization Mode	VV + VH	Dual-polarization Sentinel-1 data
Data Partitioning	Number of Subsets	5	Divided by temporal segments
Images per Subset	Number of SAR scenes per subset	11	Automatically adjusted by time coverage
SHP Identification	Homogeneous pixel window size	7 × 7	Ensures statistical homogeneity
Coherence Matrix	Type of Coherence Matrix	Total Power (TP) TSTP coherence matrix	Constructed using all polarimetric channels
Data Compression	Subspace Dimension (Compression Rank)	1	Assumes single dominant scattering
Phase Optimization	Optimization Algorithm	Expectation-Maximization Iteration (EMI) + EVD	Based on maximum likelihood estimation
Sequential Estimation	Estimator Type	Sequential Estimator	Recursive processing between subsets
Phase Referencing	Subset-wise Phase Linking Strategy	Unified dynamic reference image strategy	Ensures temporal consistency
Filtering	Temporal Homogeneous Filtering	Enabled	Enhances phase quality in low coherence areas

**Table 2:** Key imaging parameters of time-series Sentinel-1 Polarimetric SAR data.

Parameter	Parameter	Description
Sensor	Sentinel-1A	ESA C-band SAR
Polarization	VV + VH	Dual-pol acquisition
Number of Images	55	2022–2024 stack
Frequency	5.405 GHz	C-band carrier
Range Spacing	2.3 m	Ground range
Azimuth Spacing	13.9 m	Along-track
Imaging Mode	IW	TOPS mode

**Table 3:** Number and spatial density of measurement points (MPs) derived from Three InSAR methods.

	Number of MPs	Spatial Density (MPs/km^2^)	Number of Images
PSI	110,653	27	55
SBAS	779,367	196	54
DSI	2,489,144	629	55

## Data Availability

The datasets used and analyzed during the current study are available from the corresponding author on reasonable request.

## Abbreviations

The following abbreviations are used in this manuscript:

SAR	Synthetic Aperture Radar
InSAR	Interferometric Synthetic Aperture Radar
DS-InSAR	Distributed Scatterer Interferometric SAR
PS-InSAR	Persistent Scatterer Interferometric SAR
SBAS	Small Baseline Subset
SETP	Subset-based Efficient Temporal Processing
EMI	Expectation–Maximization Inversion
SETP-EMI	Sequential Estimation and Total Power-enhanced EMI
TP	Total Power (Polarimetric Coherency Matrix)
SLC	Single-Look Complex Image
DEM	Digital Elevation Model
LOS	Line of Sight
GACOS	Generic Atmospheric Correction Online Service
RMSE	Root Mean Square Error
STD	Standard Deviation
VV/VH	Vertical Transmit–Vertical Receive/Vertical Transmit–Horizontal Receive
MPs	Measurement Points (Coherent Scatterer Points)
AOI	Area of Interest
